# Value of serum CEA and CA19–9 dynamic changes and trajectory modeling in prognosis assessment for non-small cell lung cancer

**DOI:** 10.3389/fonc.2026.1808730

**Published:** 2026-07-27

**Authors:** Jing Huang, Tianyun Qiao, Huijie Meng, Fujun Duan, Shaen Li, Liping Peng, Yuyuan Liu

**Affiliations:** 1 Department of Cardiothoracic Surgery, Air Force Hospital of the Southern War Zone of the Chinese People’s Liberation Army, Guangzhou, China; 2 Department of Pulmonary and Critical Care Medicine, Air Force Hospital of the Southern War Zone of the Chinese People’s Liberation Army, Guangzhou, China; 3 Department of Outpatient, Air Force Hospital of the Southern War Zone of the Chinese People’s Liberation Army, Guangzhou, China; 4 Department of Respiratory Medicine, The First Affiliated Hospital of Changsha Medical University, Changsha, China

**Keywords:** CA19-9, CEA, non-small cell lung cancer, predictive model, trajectory fitting

## Abstract

**Objective:**

To investigate the value of dynamic changes and trajectory fitting of serum carcinoembryonic antigen (CEA) and carbohydrate antigen 19-9 (CA19-9) in the prognosis of non-small cell lung cancer (NSCLC).

**Methods:**

A retrospective analysis was conducted on 244 NSCLC patients treated in our hospital from January 2019 to December 2022. CEA and CA19–9 levels were collected before treatment and at 14 days, 28 days, 3 months, and 6 months post-treatment. Latent class growth models were used to analyze the change trajectories of the CEA and CA19–9 levels. Independent prognostic factors affecting overall survival (OS) in patients with NSCLC were identified using Cox regression analysis. A Nomogram model was developed to predict survival rates, with the classification performance evaluated using calibration curves and the area under the time-dependent ROC curve (AUC).

**Results:**

Both CEA and CA19–9 trajectories in NSCLC patients were categorized into two distinct patterns: a continuously decreasing pattern and a decreasing-then-increasing pattern. The Results from the multivariate COX regression and nomogram indicated that advanced age, smoking, advanced TNM stage, squamous cell carcinoma, and decreased CEA or CA19–9 levels before reaching stable state were factors associated with reduced OS in patients with NSCLC. The AUC values of the ROC curves for the 1-, 2-, and 3-year survival rates in the training set were 0.733, 0.863, and 0.840, respectively. The AUC values for the test set were 0.769, 0.726, and 0.760, respectively.

**Conclusion:**

Advanced age, smoking history, high TNM stage, squamous cell carcinoma, initial decrease followed by an increase in CEA, and initial decrease followed by an increase in CA19–9 are risk factors for reduced OS in patients with NSCLC. The clinical application of these factors enables the early identification of high-risk patients, facilitating early detection and intervention, and provides valuable data references for improving the prognosis of patients with NSCLC.

## Introduction

According to GLOBOCAN 2025 data, lung cancer ranks second globally in terms of incidence and remains the leading cause of cancer-related deaths, with a mortality rate as high as 3.2 per 100,000 individuals. In China, lung cancer is the most common malignant tumor in terms of incidence and mortality. Statistics show that there were 1.176 million new lung cancer cases in 2024, representing a significant cancer burden. Among lung cancers, non-small cell lung cancer (NSCLC) is the most common type, accounting for approximately 85% of all lung cancers (1, 2). NSCLC often develops insidiously, with nonspecific early symptoms. Most cases are inoperable and metastatic at the time of initial diagnosis. Currently, chemotherapy-based comprehensive treatment remains the mainstay of clinical management; however, the overall prognosis remains poor (3, 4). Tumor markers (TM) are substances present in bodily tissues and fluids that reflect tumor presence and growth. They are either synthesized and secreted by tumor cells or generated by the body in response to tumor cells. Tumor markers can be used not only for early tumor diagnosis but also to reflect disease progression and prognosis (5, 6). Carcinoembryonic antigen (CEA) is a polysaccharide-rich protein complex expressed on the surface of cancer cells derived from endodermal cell differentiation, making it detectable in various malignancies (7, 8). CA19–9 is a mucin-type glycoprotein whose expression levels may correlate with NSCLC histological subtypes, clinical staging, treatment response, and prognosis (9, 10). However, studies investigating the trajectory of CEA and CA19–9 levels before and after treatment remain scarce. Therefore, this retrospective analysis investigated the association between CEA and CA19–9 trajectory changes and the prognosis of patients with NSCLC, aiming to provide data-driven evidence for prognostic evaluation.

## Materials and methods

### Baseline data

A retrospective analysis was conducted on 244 patients with NSCLC who were treated in our hospital from January 2019 to December 2022. Follow-up continued until December 2025, with a median follow-up duration of 47.50 months. Overall survival (OS) was defined as the time (in months) from diagnosis to death or the last follow-up visit (11). Follow-up methods included telephone follow-up, electronic medical record reviews, and outpatient follow-up visits. The inclusion criteria were as follows: (1) histopathologically confirmed NSCLC; (2) complete clinical and pathological data; and (3) achievable follow-up to determine overall survival. The exclusion criteria were as follows: (1) concurrent chronic respiratory diseases such as bronchial asthma or chronic obstructive pulmonary disease; (2) severe systemic diseases or significant impairment of liver, kidney, cardiac, hematologic, or pulmonary function; (3) concurrent other malignancies; (4) acute or chronic infections or antibiotic use within 1 month prior to NSCLC diagnosis; and (5) Patients with a post-treatment survival time of less than 6 months or those loss to follow-up. This research was approved by the Ethic Committee of the Air Force Hospital of the Southern War Zone of the Chinese People’s Liberation Army (AFHLL-2026021). It was carried out in compliance with the Declaration of Helsinki.

### Treatment approach

All patients received first-line standard treatment based on the National Comprehensive Cancer Network (NCCN) guidelines (12), European Society for Medical Oncology (ESMO) guidelines (13), and the Chinese Medical Association’s Clinical Diagnosis and Treatment Guidelines for Lung Cancer (2021 Edition) (14). This included standard first-line therapies, such as curative surgery, platinum-based doublet chemotherapy, anti-angiogenic therapy combined with chemotherapy, and immunotherapy combined with chemotherapy. All patients received at least two cycles of standard treatment.

### Observation indicators

1. General Information: Patient demographic and clinical characteristics were collected, including sex, age, body mass index (BMI, kg/m²), comorbidities (hypertension and diabetes), smoking history, alcohol consumption history, treatment modality (radical surgery, neoadjuvant therapy, or chemotherapy), TNM stage, tumor differentiation grade, and histological subtype.
2. Serum Carcinoembryonic Antigen (CEA) and Carbohydrate Antigen 19-9 (CA19-9)Measurement:

A 3 mL fasting venous blood sample was collected from each patient and centrifuged at 3,000 rpm for 15 min at 4 °C. The separated serum was used to determine CEA (Reference range: ≤5 ng/ml) and CA19–9 levels (Reference range: <37 U/mL) using a seven-tumor-marker detection kit based on microarray chemiluminescent immunoassay (100 tests/kit; Jiangsu Sanlian Bioengineering Co., Ltd.; National Medical Device Registration No. 20153400234). Serum biomarker levels were measured at baseline (before treatment) and at 14 days, 28 days, 3 months, and 6 months after treatment.

### Statistical analysis

A latent class growth model was fitted using Mplus 8.3 to analyze the trajectory changes of CEA and CA19–9 levels in patients with NSCLC, with different groups named based on the trajectory slopes and intercepts. Data analysis was performed using SPSS version 27.0. Normally distributed continuous variables are expressed as x ± SD, and intergroup comparisons were conducted using independent samples t-test. Count data are presented as [n (%)], with intergroup comparisons performed using *χ*² tests. Ordinal data were analyzed using nonparametric tests. Using R, 70% of the patient data were selected as the training set, with the remaining 30% as the test set. Cox regression analysis was performed to identify independent prognostic factors for patients with NSCLC in the training set, and a nomogram model was constructed. The predictive performance of the model was evaluated using calibration curves and the area under the curve (AUC) of the receiver operating characteristic (ROC) curve. *P* < 0.05 was considered statistically significant.

## Results

### Latent categories of CEA and CA19–9 trajectories in NSCLC patients

Latent category growth models were constructed to describe the average trends of CEA and CA19–9 changes over one year in patients with NSCLC. The specific fitting metrics for the CEA and CA19–9 trajectory models are shown in **Tables 1** and **2**, respectively. In the latent class growth models for CEA and CA19–9 levels, when three classes were considered, the log-maximum likelihood ratio (LMR) was non-significant, and the class proportion was <10%. Therefore, two latent classes were retained for both markers.

**Table 1:** Growth model fitting metrics for CEA subclasses in NSCLC patients.

AIC	BIC	aBIC	Entropy	LMR	BLRT	Ratio (%)
7908.940	7954.403	7913.194	–	–	–	1
7902.942	7937.914	7906.215	0.599	<0.001	<0.001	19.33/80.67
7897.215	7921.695	7899.506	0.616	0.498	1.000	19.33/77.17/3.50

**Table 2:** Growth model fitting metrics for CA19–9 subclasses in NSCLC patients.

AIC	BIC	aBIC	Entropy	LMR	BLRT	Ratio (%)
11232.209	11256.689	11234.500	–	–	–	1
11211.361	11246.333	11214.634	0.657	0.010	<0.001	63.01/36.99
11213.785	11259.248	11218.040	0.789	0.174	0.325	1.23/59.84/38.93

### Trajectories of CEA in different latent categories among NSCLC patients

**Figure 1:**
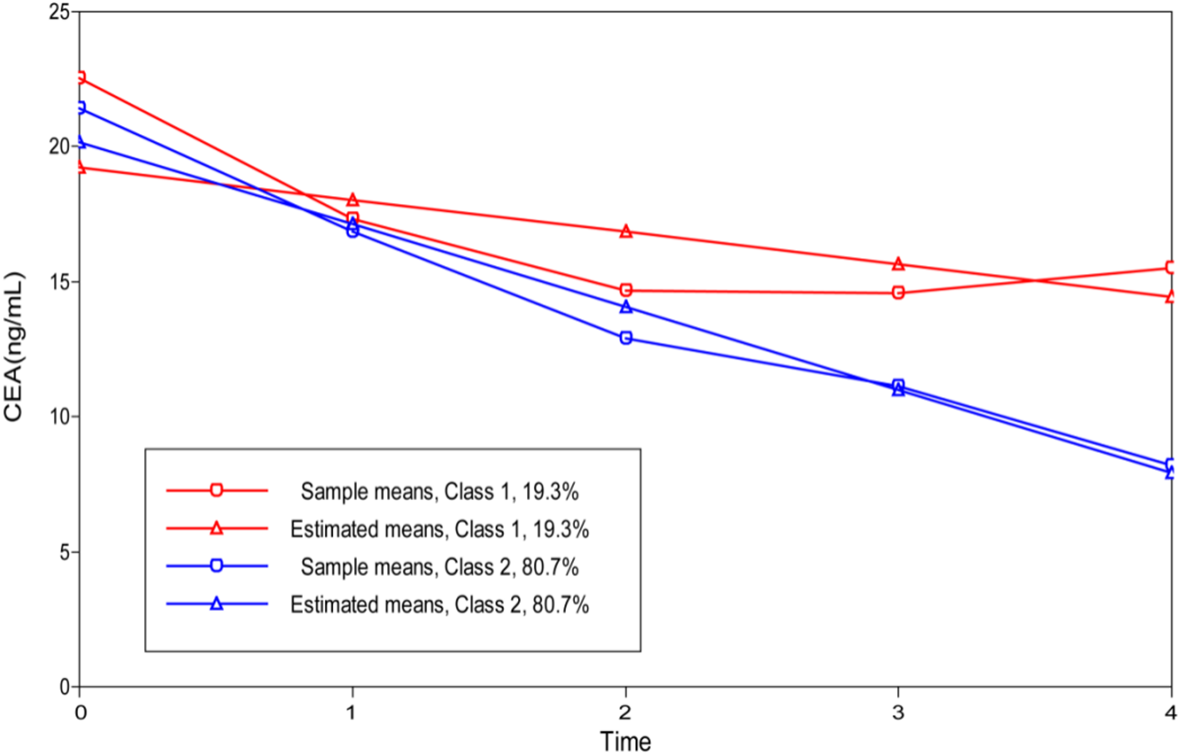
Developmental trajectory diagram of CEA subcategories in NSCLC patients. 0: Before treatment; 1: 14 days after treatment; 2: 28 days after treatment; 3: 3 months after treatment; 4: 6 months after treatment.

The latent class growth model identified two trajectory categories for CEA over time: Class 1 (decrease before stable group, n=47, 19.33%) with intercept (*I*) of 19.216 (*P* < 0.001) and slope (*S*) showing a significant downward trend (*S* = -1.191, *P* = 0.030); Class 2 (decreasing group, n=197, 80.67%), with an intercept (*I*) of 20.177 (*P* < 0.001) and a significantly decreasing slope (*S* = -3.065, *P* < 0.001). No statistically significant difference was observed in the baseline CEA levels between the categories (*P*>0.05) (**Figure 1**).

### Trajectories of CA19–9 in different latent categories among NSCLC patients

**Figure 2:**
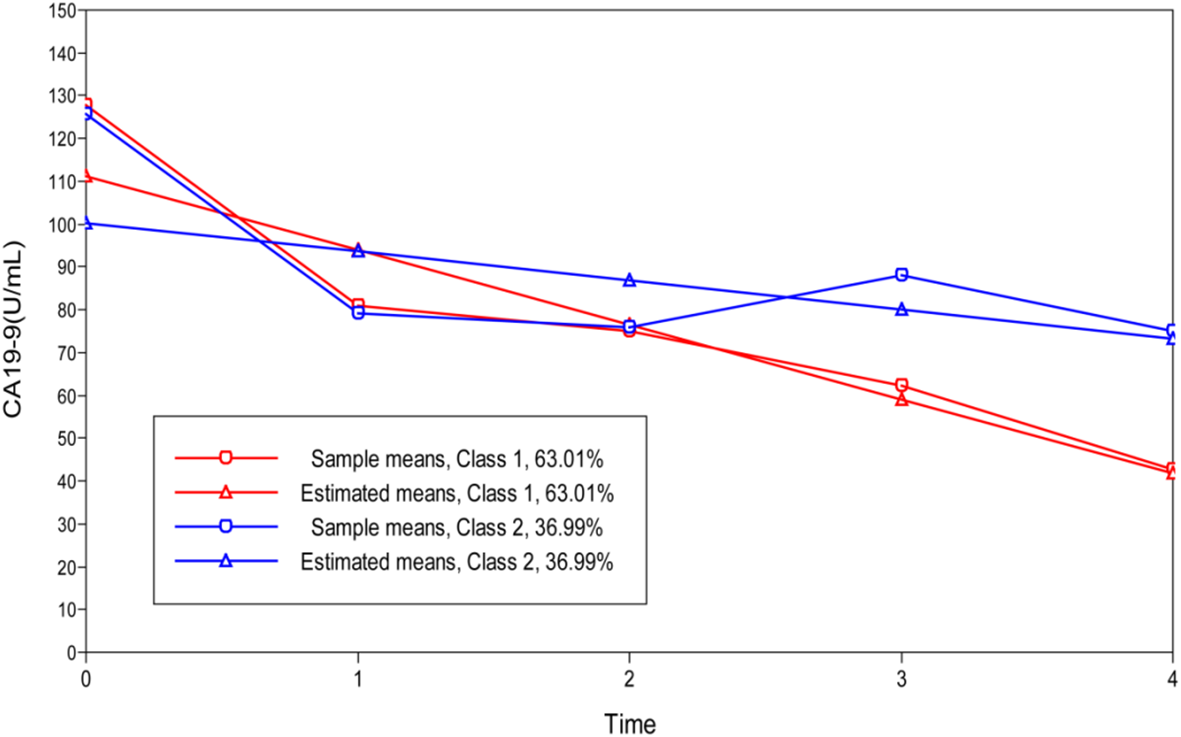
Developmental trajectory diagram of CA19–9 subcategories in NSCLC patients. 0: Before treatment; 1: 14 days after treatment; 2: 28 days after treatment; 3: 3 months after treatment; 4: 6 months after treatment.

The latent class growth model identified two trajectory categories for CA19–9 over time: Class 1 (Decline Group, n=154, 63.01%), with *I* = 111.255 (*P* < 0.001) and a significant downward trend in *S* (*S* = -17.374, *P* < 0.001); Class 2 (Decrease before stable group, n=90, 36.99%), with *I* = 100.322 (*P* < 0.001) and S showing a significant downward trend (*S* = -6.774, *P* < 0.001). No statistically significant difference was observed in the baseline CA19–9 levels between the categories (*P*>0.05) (**Figure 2**).

### Clinical baseline characteristics of 244 patients

This study included 244 patients with NSCLC. At the end of the follow-up, patients were divided into deceased (n=134) and survivor groups (n=110) based on their survival status. Baseline characteristic comparisons revealed that the deceased group was significantly older than the survivor group. Significant differences were also observed in smoking history, TNM staging, tumor subtype, CEA trajectory, and CA19–9 trajectory between the groups (*P* < 0.05). No significant differences were observed in the other indicators (*P* > 0.05) (**Table 3**).

**Table 3:** Clinical baseline characteristics of 244 patients by survival status.

Characters	Death group (n=134)	Survival group (n=110)	*t/x^2^/Z*	*P*
Sex			0.959	0.327
Male	73 (54.48)	53 (48.18)		
Female	61 (45.52)	57 (51.82)		
Age (years)	65.15 ± 8.99	60.05 ± 7.55	4.727	<0.001
BMI (kg/m^2^)	23.02 ± 3.60	22.66 ± 3.26	0.801	0.424
Hypertension			0.003	0.960
Yes	69 (51.49)	57 (51.82)		
No	65 (48.51)	53 (48.18)		
Diabetes			0.276	0.599
Yes	62 (46.27)	55 (50.00)		
No	71 (52.99)	55 (50.00)		
Smoking history			7.606	0.006
Yes	81 (60.45)	47 (42.73)		
No	53 (39.55)	63 (57.27)		
Alcohol consumption history			0.513	0.474
Yes	56 (41.79)	51 (46.36)		
No	78 (58.21)	59 (53.64)		
Treatment modality			2.410	0.300
Curative surgery	39 (29.10)	42 (38.18)		
Neoadjuvant therapy	50 (37.32)	38 (34.55)		
Chemotherapy	45 (33.58)	30 (27.27)		
TNM stage			-2.773	0.006
I	25 (18.66)	29 (26.36)		
II	21 (15.67)	31 (28.18)		
III	45 (33.58)	27 (24.55)		
IV	43 (32.09)	23 (20.91)		
Degree of differentiation			0.112	0.738
Low	82 (61.19)	65 (59.09)		
Middle/High	52 (38.81)	45 (40.91)		
Tumor Classification			7.214	0.007
Adenocarcinoma	66 (49.25)	73 (66.36)		
Squamous cell carcinoma	68 (50.75)	37 (33.64)		
Baseline CEA (ng/mL)	20.65 (16.30,27.03)	20.55 (15.80,27.63)	-0.192	0.847
Baseline CA19-9 (U/mL)	129.00 (115.25,141.00)	122.50 (110.75,140.00)	-1.532	0.125
CEA Trajectory			4.076	0.043
Decrease	102 (76.12)	95 (86.36)		
Decrease before stable	32 (23.88)	15 (13.64)		
CA19–9 Trajectory			7.950	0.005
Decrease	74 (55.22)	80 (72.73)		
Decrease before stable	60 (44.78)	30 (27.27)		

### Multivariate Cox regression analysis of overall survival in patients with NSCLC

Using survival status (1=death, 0=alive) as the dependent variable, OS as the time variable, and patient age (assigned original value), smoking history (1=Yes, 0=No), TNM staging (1=I, 2=II, 3=III, 4=IV), tumor subtype (1=adenocarcinoma, 2=squamous cell carcinoma), CEA traje(1=decreasing, 2=decrease before stable), and CA19–9 trajectory (1=decreasing, 2=Decrease before stable) as independent variables. The results indicated that age (HR = 1.030, 95% CI: 1.010-1.051), smoking history (HR = 1.750, 95% CI: 1.226-2.498), TNM stage, tumor classification (HR = 1.721, 95% CI: 1.203-2.464), CEA trajectory (HR = 1.551, 95% CI: 1.022-2.354), and CA19–9 trajectory (HR = 1.534, 95% CI: 1.069-2.202) were independent factors affecting OS in patients with NSCLC (*P* < 0.05) (**Table 4**). To test the proportional-hazards assumption of the Cox regression model, this study further calculated the Schoenfeld residuals for each covariate and conducted a global test of the model as a whole. The results showed that the Cox proportional-hazards model satisfied the proportional-hazards assumption (global *P* = 0.575; all covariates *P*>0.05).

**Table 4:** Multivariate cox regression analysis of factors affecting overall survival in NSCLC patients.

Characters	B	SE	*Wald*	*P*	HR	95% CI
Age	0.030	0.010	8.503	0.004	1.030	1.010-1.051
Smoke	0.559	0.182	9.494	0.002	1.750	1.226-2.498
TNM						
I					1.000	Reference
II	0.519	0.332	2.454	0.117	1.681	0.878-3.220
III	0.611	0.295	4.296	0.038	1.842	1.034-3.283
IV	0.969	0.315	9.440	0.002	2.634	1.420-4.886
Tumor Classification	0.543	0.183	8.807	0.003	1.721	1.203-2.464
CEA Trajectory	0.439	0.213	4.251	0.039	1.551	1.022-2.354
CA19–9 Trajectory	0.428	0.184	5.387	0.020	1.534	1.069-2.202

### Construction of a survival prediction nomogram model for patients with NSCLC

**Figure 3:**
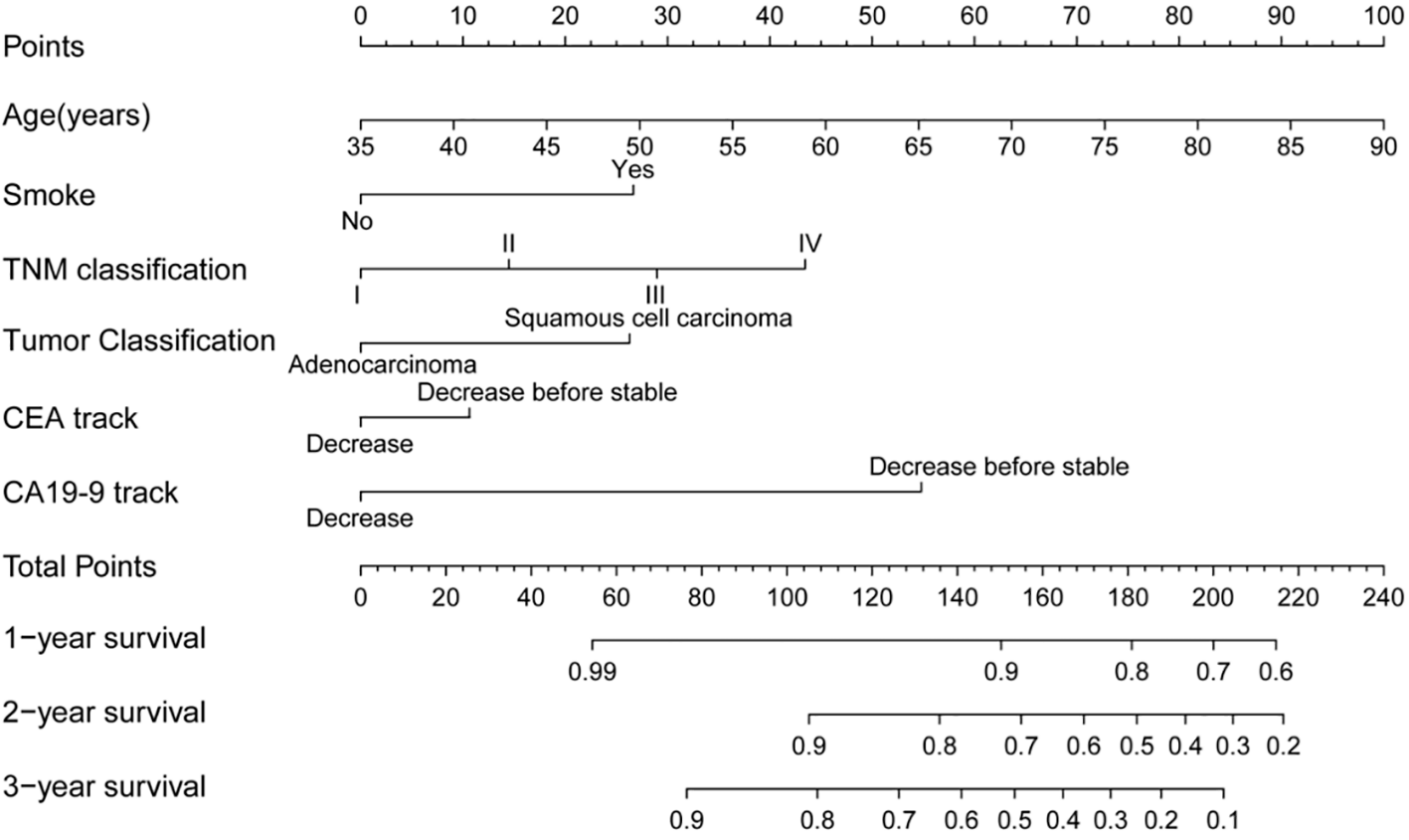
Nomogram model for survival rate prediction for NSCLC patients.

**Figure 4:**
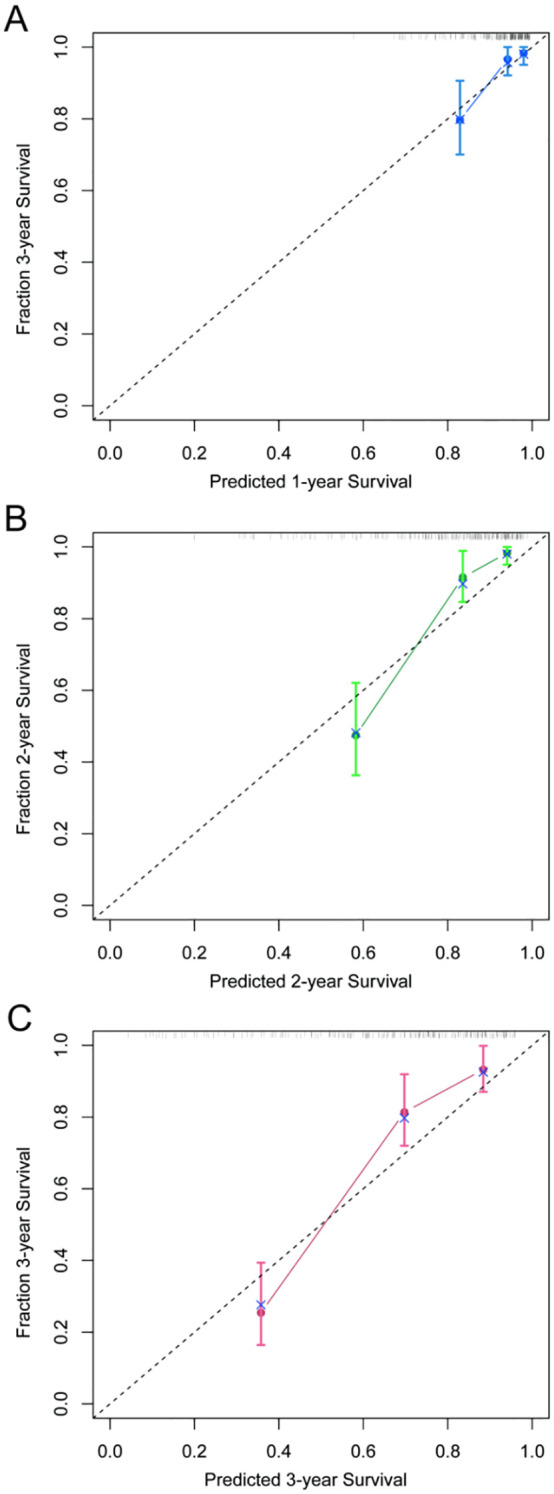
Calibration curves for survival rates of NSCLC patients in the training set. **(A)** 1-year; **(B)** 2-year; **(C)** 3-year.

**Figure 5:**
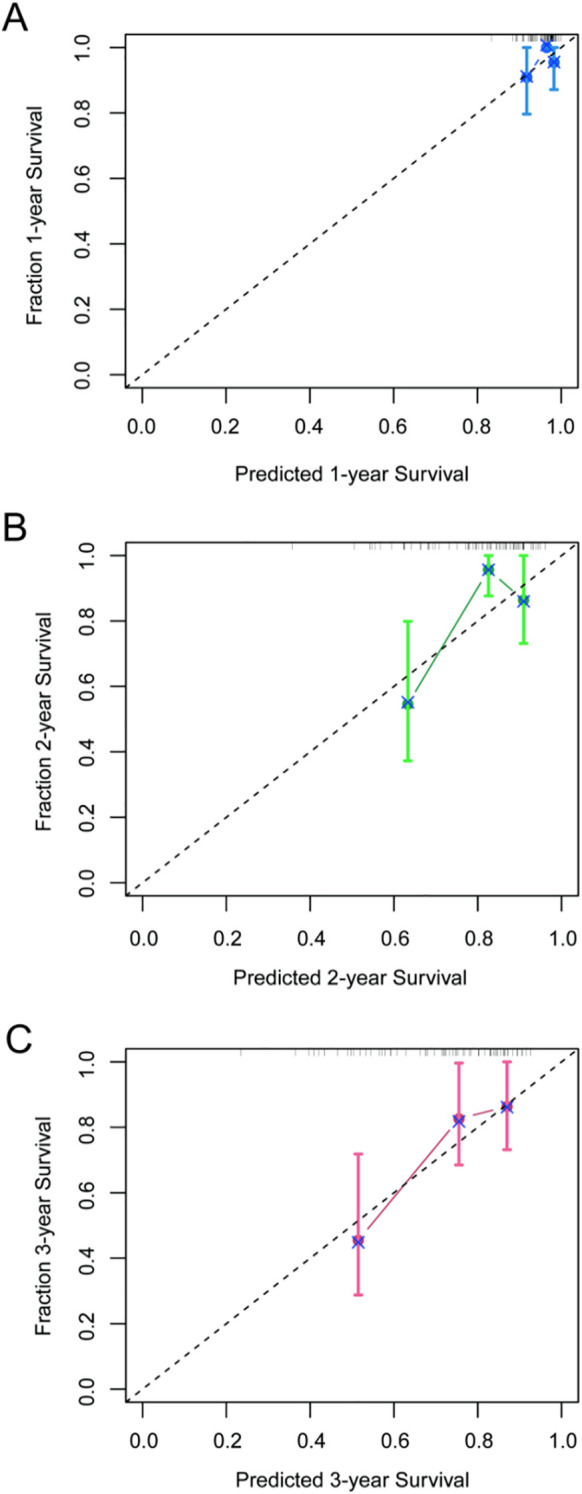
Calibration curves for survival rates of NSCLC patients in the test set. **(A)** 1-year; **(B)** 2-year; **(C)** 3-year.

Variables with P < 0.05 in the univariate analysis were included in the Cox regression analysis. Independent prognostic factors identified in the multivariate Cox regression analysis (Age, Smoking History, TNM Stage, Tumor Classification, CEA Track, and CA19–9 Track) were incorporated into a survival prediction nomogram model for patients with NSCLC (**Figure 3**). The results indicated that advanced age, smoking history, high TNM stage, squamous cell carcinoma, CEA levels that experience decrease before stabilizing, and CA19–9 levels that experience decrease before stabilizing were risk factors for reduced OS in patients with NSCLC. The calibration curves for the training and testing sets, along with the time-dependent ROC curves at 1, 2, and 3 years, demonstrated good agreement between the calibration curves and the ideal reference line (**Figures 4**, **5**).

### ROC curves for the nomogram model

**Figure 6:**
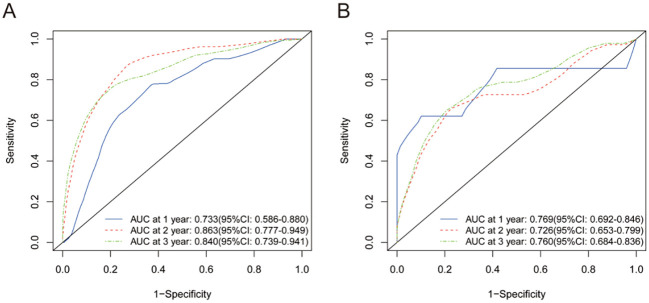
ROC curves for predicting 1-year, 2-year, and 3-year survival rates in NSCLC patients using the column chart. **(A)** Train set; **(B)** Test set.

**Figure 7:**
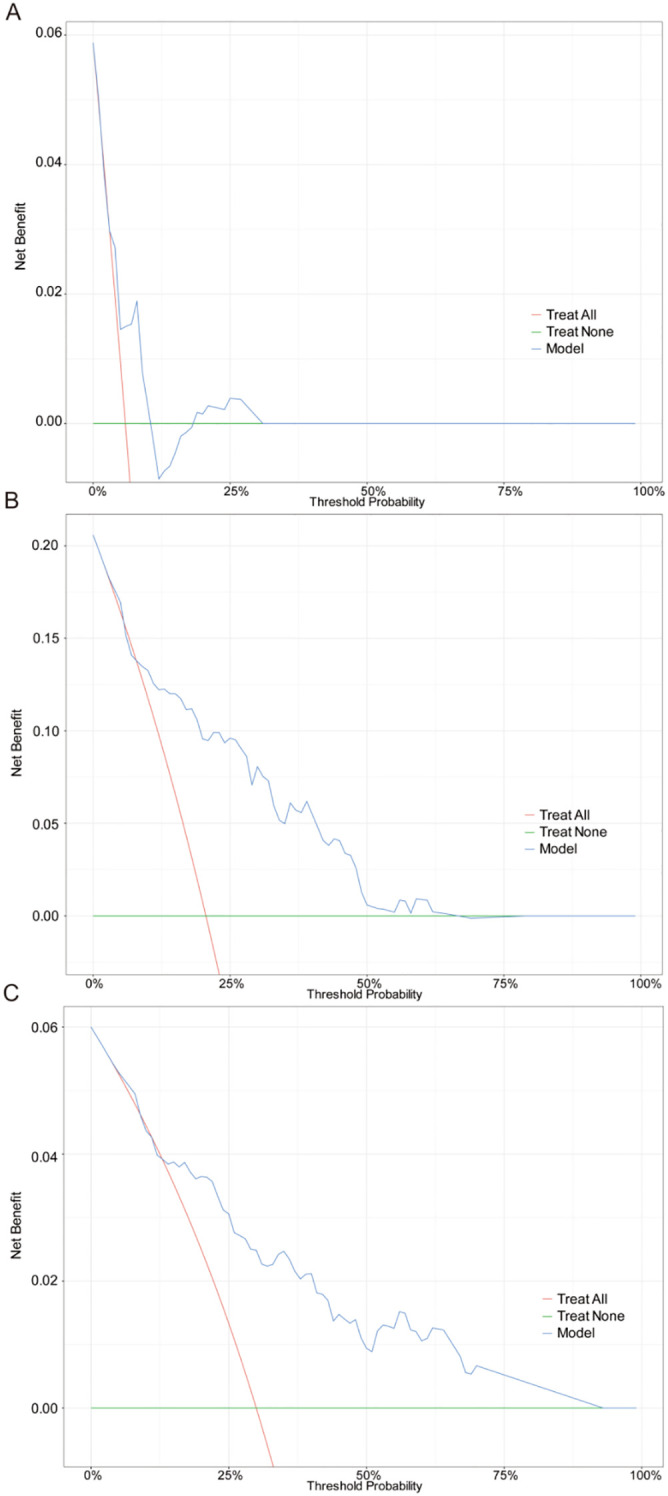
DCA curve for survival rates of NSCLC patients in the train set. **(A)** 1-year; **(B)** 2-year; **(C)** 3-year.

**Figure 8:**
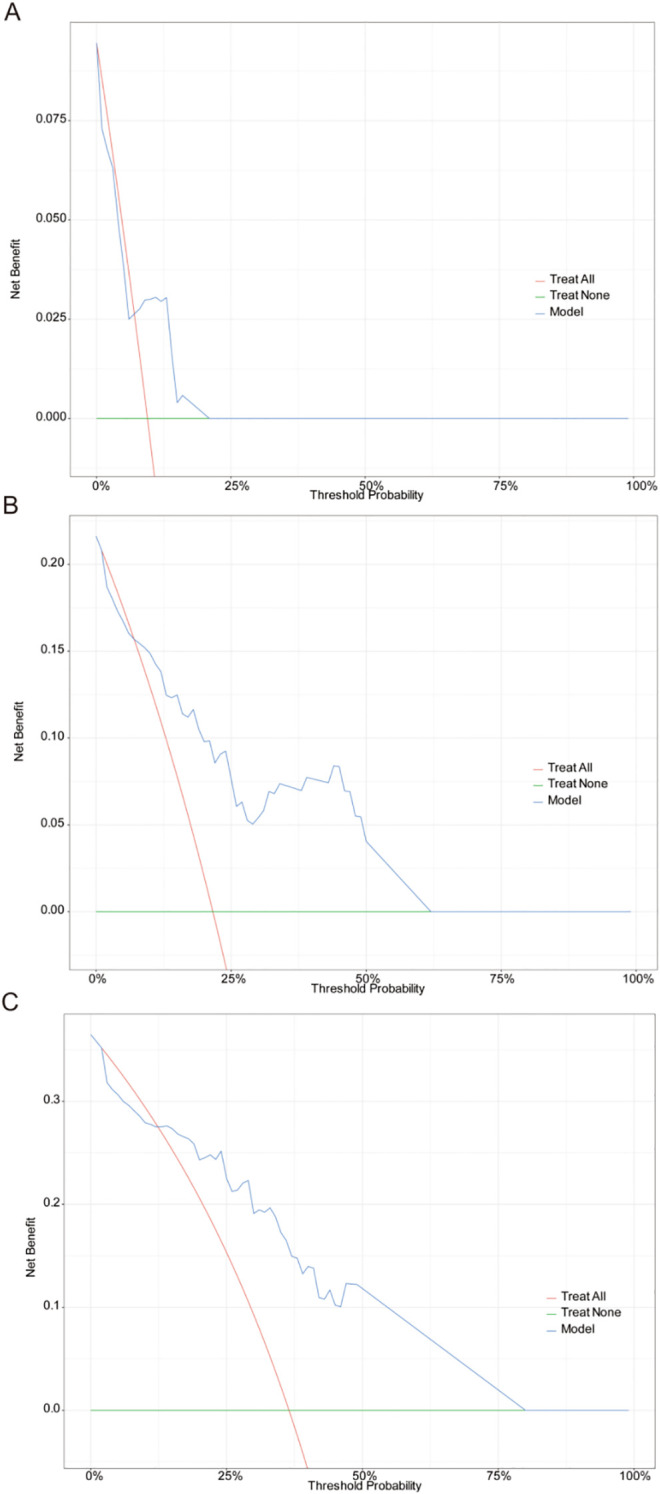
DCA curve for survival rates of NSCLC patients in the test set. **(A)** 1-year; **(B)** 2-year; **(C)** 3-year.

The AUC values of the ROC curves for the training set’s 1-year, 2-year, and 3-year survival rates were 0.733, 0.863, and 0.840, respectively (**Figure 6A**). The areas under the ROC curves for the test set’s 1-year, 2-year, and 3-year survival rates were 0.769, 0.726, and 0.760 (**Figure 6B**), indicating that the model possessed a good discriminatory ability. The DCA curves for 1-year, 2-year, and 3-year survival rates further demonstrated that the model yielded positive clinical net benefits in both the training and test sets (**Figures 7**, **8**).

### External validation

Data from 125 patients with NSCLC admitted to our hospital between January 2023 and December 2024 were collected to externally validatethe 1-year survival rate. The accuracy, sensitivity, and specificity of the confusion matrix were 88.00%, 65.22%, and 93.14%, respectively (**Table 5**).

**Table 5:** External validation confusion matrix and metrics for 1-year survival (%).

Model	Predicted	Reference		Total	Indicator	%
		Death	Survival			
Nomogram	Death	15	8	23	Accuracy	88.00
	Survival	7	95	102	Sensitivity	65.22
Total		22	103	125	Specificity	93.14

## Discussion

In China, lung cancer remains one of the cancers with high incidence and mortality rates. NSCLC accounts for the largest proportion of lung cancer cases, and most patients are diagnosed at an advanced stage, missing the optimal treatment window. In recent years, the number of new lung cancer diagnoses in China has continued to increase (15). To improve the quality of life and clinical outcomes for patients with NSCLC, clinicians and patients urgently need a comprehensive assessment system to identify high-risk patients and predict prognosis, thereby providing valuable data references for cancer diagnosis and treatment plans.

This study found that the trajectories of CEA and CA19–9 in patients with NSCLC can be categorized into two distinct patterns: a decrease-then-stable pattern and a continuous-decrease pattern. Research indicates that CEA and CA19–9 levels in patients with NSCLC are significantly higher than those in healthy controls, and the combined diagnosis of both markers may aid in NSCLC screening (16). This study found that CEA and CA19–9 levels in all 244 patients prior to treatment exceeded the normal range, potentially related to the pathological stage of the disease. Within 28 days post-treatment, all patients exhibited a downward trend in CEA and CA19–9 levels, likely due to the reduced tumor burden following the standard therapy. However, some patients demonstrated elevated CEA and CA19–9 levels 3 months post-treatment. This may indicate poor short-to medium-term treatment efficacy in these patients, potentially suggesting recurrence or metastasis and indicating a poor prognosis. Based on these findings, this study employed the distinct trajectories of CEA and CA19–9 levels as independent variables. Combined with other clinical data, multivariate Cox regression analysis was conducted to assess OS in patients with NSCLC. Variables with P < 0.05 were incorporated into the nomogram model. The results indicated that advanced age, smoking history, high TNM stage, squamous cell carcinoma, CEA levels that decrease before stabilization, and CA19–9 levels that edecrease before stabilization were risk factors for reduced OS in patients with NSCLC. Older patients often have comorbidities, and declining organ function with age further contributes to reduced OS (17–19). Research indicates that environmental factors and lifestyle choices are risk factors for lung cancer. Statistics show that smoking accounts for 85% to 90% of these factors, with the risk of developing lung cancer increasing with longer smoking duration and higher daily cigarette consumption (20, 21). Previous studies have demonstrated that patients with higher TNM staging exhibit a poorer prognosis. The findings of this study are consistent with this observation. This maybe because a higher TNM stage indicates tumor spread and metastasis, resulting in poorer treatment efficacy and prognosis (22, 23). Squamous cell carcinoma primarily arises from chronic irritation and damage to bronchial mucosal epithelial cells, leading to basal cell squamous metaplasia, atypical hyperplasia, or dysplasia, which subsequently progresses to malignancy (24). Squamous cell carcinoma tissue is more prone to degeneration and necrosisand exhibits reduced sensitivity to radiotherapy and chemotherapy. This increases the risk of poor prognosis and reduced OS following treatment. Studies have indicated that combining immunotherapy with chemotherapy can improve survival rates in patients with advanced squamous NSCLC without altering targetable genes (25). Previous studies have demonstrated a positive correlation between elevated postoperative CEA levels and recurrence risk and mortality (26), consistent with our findings. The decrease before stability in CEA and CA19–9 levels suggests potential recurrence or metastasis in NSCLC post-treatment, often necessitating readmission for renewed therapy. Both chemotherapy and immunotherapy impose additional physiological burdens (27), thereby reducing OS in patients with NSCLC. The results of this study showed that the AUC values of the ROC curves for predicting 1-, 2-, and 3-year survival in the training cohort of the NSCLC nomogram model were 0.733, 0.863, and 0.840, respectively, whereas the corresponding AUC values in the validation cohort were 0.769, 0.726, and 0.760, respectively. The results indicate that the model demonstrates acceptable discriminatory power and can serve as an exploratory prognostic tool to assist clinicians in developing personalized treatment plans.

In summary, advanced age, smoking history, high TNM stage, squamous cell carcinoma, a decrease-then-stable CEA trajectory, and a decrease-then-stable CA19–9 trajectory were identified as risk factors for reduced OS in patients with NSCLC. The clinical application of these factors enables the early identification of high-risk patients, facilitating early detection and intervention, and provides valuable data references for improving the prognosis of patients with NSCLC. However, this study has certain limitations. It was a single-center, retrospective study with a small sample size and a single source of data, and there are also confounding factors that were not included in the study (such as ECOG score, molecular markers, metastatic burden, etc.), which may have introduced selection bias. Furthermore, the entropy values of the CEA trajectory models identified by our institute to date are slightly low, and the models we have developed are exploratory prognostic tools that cannot support clinical decision-making. Therefore, future prospective multicenter studies with larger sample sizes are warranted to provide additional evidence for further optimizing personalized nursing care pathway protocolsand refining prognostic risk prediction models for t patients with NSCLC.

## Conclusion

Older age, a history of smoking, advanced TNM stage, squamous cell carcinoma, a decrease-then-stable CEA trajectorya d, and Decrease-then-stable CA19–9 trajectory were identified as risk factors for reduced overall survival in patients with non-small cell lung cancer. The application of these factors in clinical practice may facilitate the early identification of high-risk patients, enabling timely detection and intervention, and may provide valuable evidence for improving the prognosis of patients with NSCLC.

## Data Availability

The original contributions presented in the study are included in the article/supplementary material. Further inquiries can be directed to the corresponding author.
